# PRRT2 Mutant Leads to Dysfunction of Glutamate Signaling

**DOI:** 10.3390/ijms16059134

**Published:** 2015-04-23

**Authors:** Ming Li, Fenghe Niu, Xilin Zhu, Xiaopan Wu, Ning Shen, Xiaozhong Peng, Ying Liu

**Affiliations:** State Key Laboratory of Medical Molecular Biology, Institute of Basic Medical Sciences, Chinese Academy of Medical Sciences, School of Basic Medicine, Peking Union Medical College, Beijing 100005, China; E-Mails: liming7654321@126.com (M.L.); niufenghe@163.com (F.N.); xlxl7280@163.com (X.Z.); wuxiaopan1984@tom.com (X.W.); 13693298675@163.com (N.S.); peng_xiaozhong@163.com (X.P.)

**Keywords:** PRRT2, glutamate, SNAP25, GRIA1

## Abstract

Paroxysmal kinesigenic choreoathetosis (PKC) is an inherited disease of the nervous system. We previously identified PRRT2 as the causative gene of PKC. However, as little is known about the function of PRRT2, elucidating its function will benefit not only PKC studies, but also many other related disorders. Here, we reveal higher levels of glutamate in the plasma of PKC patients and the culture medium of neurons following knock-out Prrt2 expression. Using double immunostaining assays we confirm Prrt2 is located at the glutamatergic neurons in accordance with its function. Our co-immunoprecipitation assays reveal mutant PRRT2 interferes with SNAP25 and GRIA1 interactions, respectively. Furthermore, using live-labeling techniques, we confirmed co-transfection with mutant PRRT2 caused an increase in GRIA1 distribution on the cell surface. Therefore, our results suggest that mutant PRRT2, probably through its weakened interaction with SNAP25, affects glutamate signaling and glutamate receptor activity, resulting in the increase of glutamate release and subsequent neuronal hyperexcitability.

## 1. Introduction

Paroxysmal kinesigenic choreoathetosis (PKC, OMIM 128200), also known as paroxysmal kinesigenic dyskinesia (PKD), was first described in 1967, and has since become the most frequently presented type of paroxysmal dyskinesias disorder [1]. It is an autosomal dominant disorder characterized by sudden and brief attacks of involuntary movement [2]. In 2011, we identified the heterozygous mutations of the proline-rich transmembrane (TM) protein 2 (PRRT2) at 16p11.2 to be responsible for PKC [3,4,5,6,7]. In another study, PRRT2 was predicted to consist of an *N*-terminal extracellular domain containing a proline-rich sequence, an *N*-glycosylation site and a *C*-terminal region with two TM domains extremely conserved among species [6]. Moreover, a study on mice revealed PRRT2 was expressed at higher levels in the cerebral cortex, hippocampus and cerebellum of the brain [4].

Few studies have explored the potential role of PRRT2. Recently, co-immunoprecipitation studies have confirmed interactions between SNAP25 and PRRT2, and revealed no expression with the truncated mutant form of PRRT2 (p.R217Pfs*8), which is considered as the most frequent mutant form found in patients. SNAP25 is a t-SNARE presynaptic protein implicated in the formation of neuronal exocytotic fusion apparatus and neurotransmitter release [8]. Many studies have detected increased SNAP25 immunoreactivity at the glutamatergic terminals [9,10,11]. In particular, specific cleavage of SNAP25 by botulinum neurotoxin E (BoNT/E) has shown to inhibit glutamate release of rat hippocampus glutamatergic neurons but have little effect on the GABAergic synapses [12]. Therefore, SNAP25 plays an important role in the regulation of excitatory amino acids (EAAs) neurotransmitter release. The dysfunction of EAA transmission, especially glutamate transmission, plays a role in many nervous diseases, including epilepsy, migraines and children with high functioning autism [13,14,15]. A recent high-resolution proteomics analysis offered another important clue into the function of PRRT2. The study identified 21 novel proteins containing PRRT2 in native α-amino-3-hydroxy-5-methyl-4-isoxazolepropionic acid (AMPA) receptor complexes [16]. The AMPA receptor is one of the ionotropic glutamate receptors consisting of four different inner subunits of GRIA1-4. Accordingly, PRRT2 is preferentially associated with the inner GRIA1 component [16].

Here, we studied the underlying mechanism for PRRT2 mutations leading to PKC. Firstly, we revealed that there were higher glutamate levels in the plasma samples of PKC patients. We then used lentivirus harboring a short-hairpin RNA (shRNA) sequence targeted to Prrt2 to infect mouse cortex neurons, which simulated the effect of truncated PRRT2. We showed an increase in glutamate levels in the culture medium of neurons after infection with shRNA-Prrt2 lentivirus, suggesting that mutant PRRT2 might affect the release of glutamate. We revealed that mutant PRRT2 interfered with SNAP25 interactions, indicating its role in the molecular mechanism. Furthermore, we proved interactions existed between PRRT2 and GRIA1, and the distribution of GRIA1 on the cell membrane increased following co-transfection with mutant PRRT2. Our results indicate that blocking the glutamate signaling pathway is a potential therapeutic strategy for PKC.

## 2. Results

### 2.1. Higher Levels of Excitatory Amino Acids (EAAs) in the Plasma of PKC Patients

**Figure 1:**
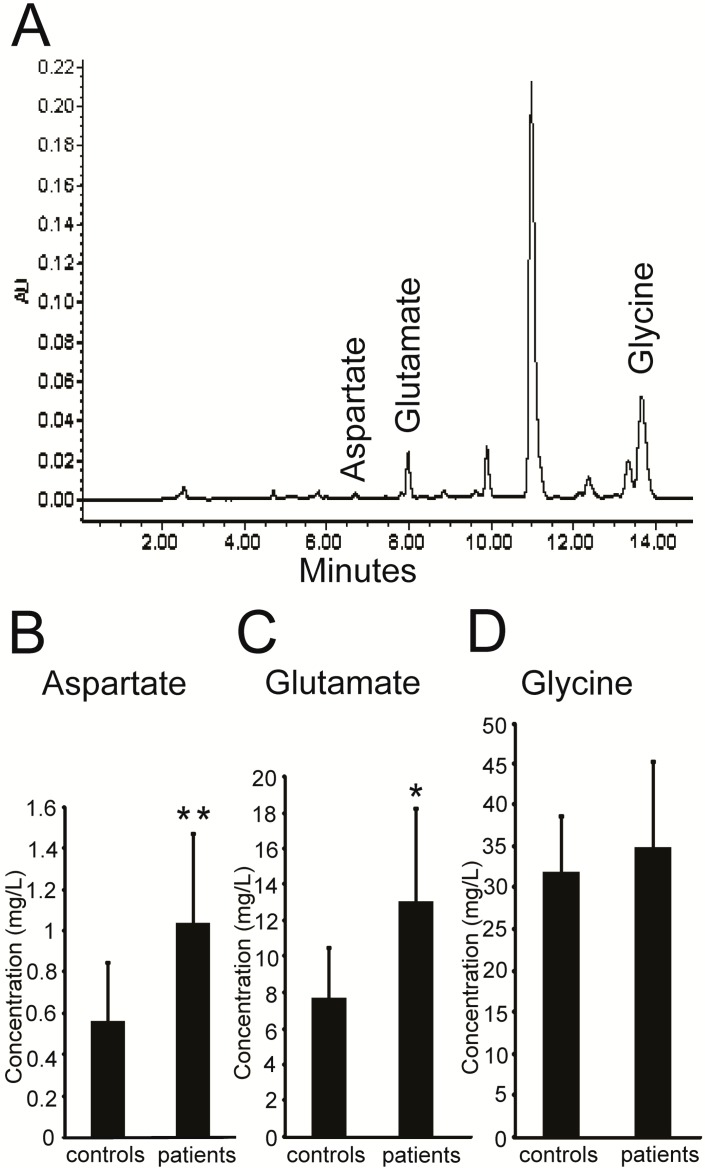
EAAs levels were higher in the plasma of PKC patients. (**A**) Representative HPLC chromatogram of three kinds of amino acids in the plasma; (**B**,**C**) The concentrations of aspartate and glutamate in the plasma from the PKC patient group (*n =* 7) were higher than those of the healthy control group (*n =* 12); (**D**) No significant difference was found in glycine levels between the two groups. Values are represented as mean ± SD, * *p* < 0.05; ** *p* < 0.01.

We first measured the levels of three amino acids (aspartate, glutamate and glycine) in the plasma of PKC patients and healthy controls via high-performance liquid chromatography (HPLC). Representative plasma chromatograms are shown in Figure 1A. We found that aspartate and glutamate concentrations were significantly higher in the plasma of PKC patients compared with healthy controls (Figure 1B,C), while glycine levels remained the same between the two groups (Figure 1D). Detailed information of the subjects from the two groups is shown in Table 1.

**Table 1:** Characteristics of the subjects.

Clinical Characteristics		PKC Patients (*n =* 7)	Healthy Controls (*n =* 12)
Mean age, year (±SD)		22.7 ± 2.5	27.3 ± 3.0
Sex	Male (%)	4 (57.1)	8 (66.7)
	Female (%)	3 (42.9)	4 (33.3)
	Age at onset, year (±SD)	13.7 ± 2.0	-

### 2.2. Knock-out Prrt2 Mice Exhibit Increased Glutamate Level in Neural Cell Culture

**Figure 2:**
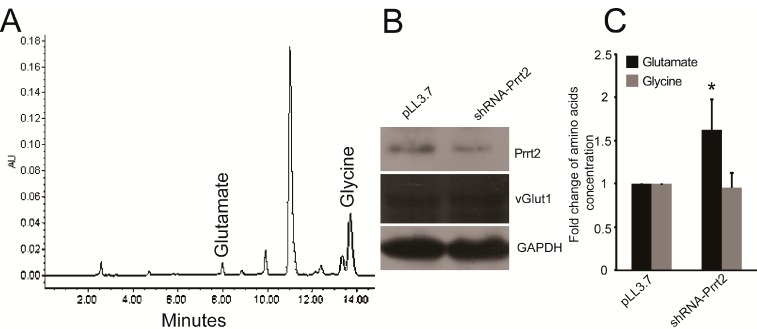
Knocking down Prrt2 increased glutamate level in the culture medium. (**A**) Representative HPLC chromatogram of glutamate and glycine in the culture medium; (**B**) Western blotting analysis was used to show the knockdown efficiency and the protein level of vGlut1 of both groups; (**C**) Summarized data (mean ± SD, *n =* 4) showed the level of glutamate and glycine detected by HPLC. * *p* < 0.05.

Considering cerebrospinal fluid (CSF) correlates positively with plasma glutamate concentrations [17,18], we performed a loss-of-function experiment using mouse cortex neurons. We generated a Prrt2-targeting shRNA lentivirus and empty pLL3.7 lentivirus from HEK293T cells. To verify gene knockdown efficiency (Figure S1, Table S1), we measured total protein extracts from mouse cortex neurons after infecting with lentivirus (Figure 2B), collecting and analyzing the culture medium by HPLC (Figure 2A). We found higher levels of glutamate in the shRNA-Prrt2 lentivirus infected group compared with the pLL3.7 control group (Figure 2C). Again, no obvious difference in glycine levels was found between the two groups (Figure 2C). Aspartate levels were too low to detect. Expression levels of vGlut1 were similar in both groups, suggesting that the uptake of glutamate is not significantly different between PKC and control patients. These results indicate PRRT2 might play an inhibitory role in glutamate release.

### 2.3. Prrt2 Is Located at the Glutamatergic Neurons

**Figure 3:**
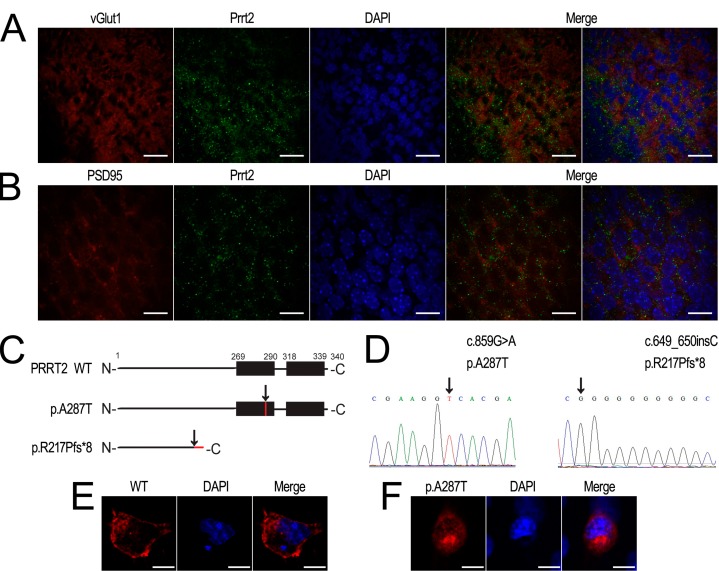
Prrt2 colocalized with presynaptic and postsynaptic markers of glutamatergic neurons of mouse cortex. (**A**,**B**) Prrt2 (green) colocalized with both vGlut1 (red) and PSD95 (red) in the cortex. Scale bar, 20 μm; (**C**) Schematic diagram illustrates the protein structure of wild type (WT) and the mutant type (p.A287T and p.R217Pfs*8) of PRRT2. Black rectangles represent two putative *C*-terminal TM domains of PRRT2. Black arrows indicate the positions of mutations. Red lines represent protein sequence produced by missense or frameshift mutations; (**D**) Sequencing maps of both c.859G>A and c.649_650insC; (**E**) COS-7 cells transfected with Flag-tagged wild type (WT) PRRT2 (red); (**F**) COS-7 cells transfected with Flag-tagged p.A287T PRRT2 (red). Scale bar, 10 μm. DAPI (blue) was used to show nuclei.

To prove the colocalization of Prrt2 with neuronal glutamatergic markers, we performed double immunostaining on frozen sections of adult mouse brain. As shown in Figure 3A, Prrt2 signals were colocalized with vGlut1 signals, the marker for presynaptic glutamatergic neuronal membrane. We also observed Prrt2 to be colocalized with the postsynaptic marker for the glutamatergic neuron, PSD-95 (Figure 3B). Therefore, we deduce Prrt2 is located at glutamatergic synapses, which is in accordance with its role in the regulation of glutamate release. To elucidate the mutant PRRT2 protein’s subcellular location, we constructed overexpressing clones of PRRT2 WT, p.R217Pfs*8 and p.A287T, where the latter was considered as the missense mutation previously reported by our lab and predicted to be damaged by SIFT (available online: http://sift.jcvi.org/) and Polyphen-2 (available online: http://genetics.bwh.harvard.edu/pph2/) [3]. The predicted structures of wild type and mutant PRRT2 are shown in Figure 3C. Sequencing maps of the mutant vectors are shown in Figure 3D. We found wild type PRRT2 mainly located at the membrane (Figure 3E), while PRRT2 with the missense mutation p.A287T lost its membrane location and was dispersed throughout the cytoplasm (Figure 3F). We did not detect any obvious signals for PRRT2 with the truncated p.R217Pfs*8 mutation (Figure 5A).

### 2.4. Interactions between PRRT2 and Its Partners

**Figure 4:**
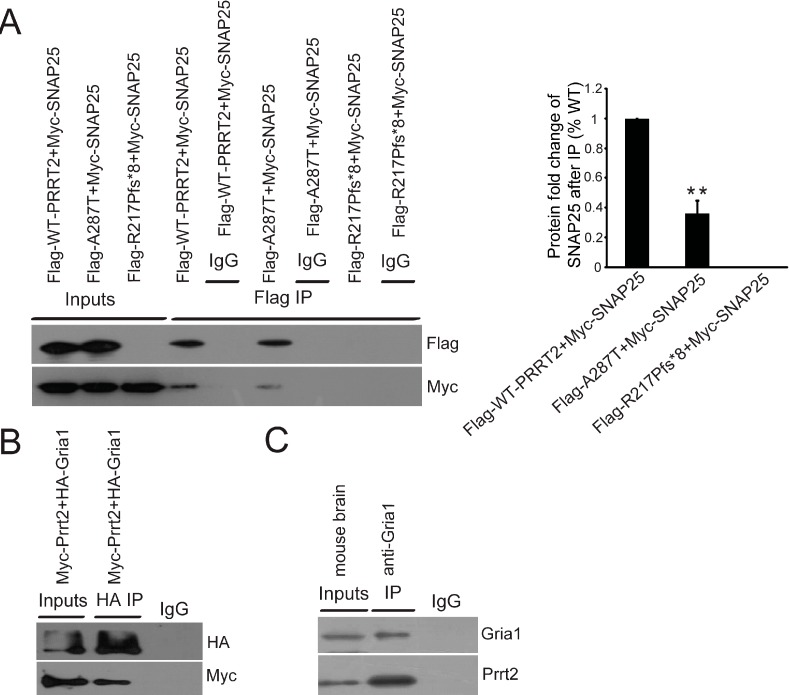

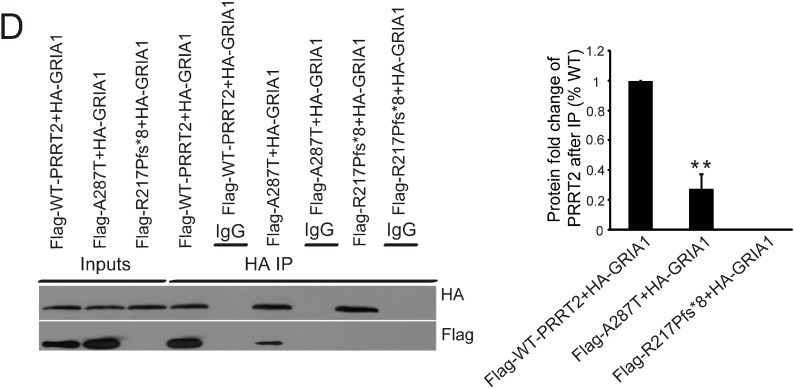
Mutant PRRT2 interfered interactions between PRRT2 and its partners. (**A**) *In vitro* co-immunoprecipitation using cell extracts from HEK293T cells co-transfected with Myc-tagged SNAP25 and Flag-tagged different forms of PRRT2. After pull-down with Flag antibody, western blotting results demonstrated interactions between SNAP25 and different forms of PRRT2. Histogram showed the fold change of SNAP25 protein level after co-immunoprecipitation with Flag antibody; (**B**) *In vitro* co-immunoprecipitation with cell extracts from HEK293T cells co-transfected with Myc-tagged Prrt2 and HA-tagged Gria1. After pull-down with HA antibody, western blotting results showed interactions between Prrt2 and Gria1; (**C**) *In vitro* immunoprecipitation of Prrt2 and Gria1 in mouse brain extract using anti-Gria1 antibody. Western blotting was used to visualize protein signals; (**D**) *In vitro* co-immunoprecipitation using cell extract from HEK293T cells co-transfected with HA-tagged GRIA1 and Flag-tagged different forms of PRRT2. After pull-down with HA antibody, western blotting results showed interactions between GRIA1 and different forms of PRRT2. Histogram shows the fold change of PRRT2 protein levels after co-immunoprecipitation with HA antibody. Values are represented as mean ± SD, *n =* 3, ** *p* < 0.01.

Because of its important role in neurosecretion, SNAP25 is the target of many regulators to modulate its neurotransmission. For example, 5-HT G protein-coupled receptors (GPCR) release G protein βγ, which directly interacts with SNAP25 and mediates presynaptic inhibition at the glutamate-releasing synapse [19]. We hypothesized that the inhibitory function of PRRT2 on the release of glutamate might similarly be due to its interaction with SNAP25. To further explore this, we performed *in vitro* co-immunoprecipitation experiments using both missense and truncated mutant PRRT2 and monitored their respective interactions with SNAP25. Consistent with previous findings, we demonstrated wild type PRRT2 interacted with SNAP25, while truncated PRRT2 (p.R217Pfs*8 mutation) failed to interact with SNAP25 [6] (Figure 4A). Compared with the wild type control, we noted a significant decrease in interactions between SNAP25 and PRRT2 with the missense mutation p.A287T (Figure 4A).

Given that GRIA1 is an important subunit of the AMPA receptor, due to its ability to facilitate synaptic insertion of AMPA receptors [20], we next performed co-immunoprecipitation assays to monitor interactions between PRRT2 and GRIA1. We first verified interactions between mouse Prrt2 and Gria1 both *in vitro* and *in vivo* (Figure 4B,C). We next revealed that, similarly to their interactions with SNAP25, there were significantly fewer interactions between missense p.A287T PRRT2 and GRIA1 compared with the wild type (Figure 4D), while no interactions at all between truncated PRRT2 (p.R217Pfs*8 mutation) and GRIA1 (Figure 4D).

### 2.5. Mutant PRRT2 Increases Distribution of GRIA1 on the Membrane

**Figure 5:**
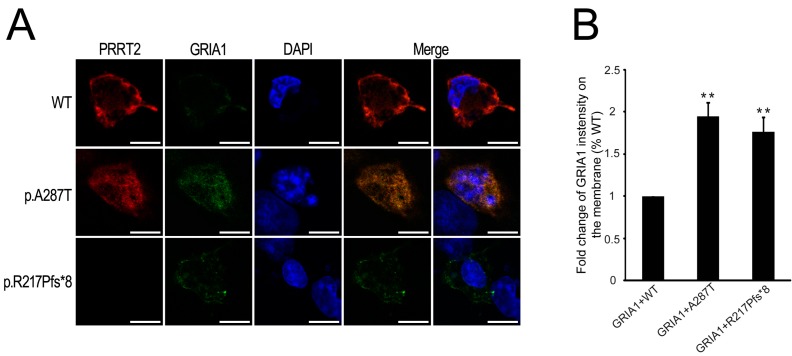
(**A**) PRRT2 limits membrane distribution of GRIA1. The first row shows Flag-tagged wild type PRRT2 (red), live-labeling of the surface HA-tagged GRIA1 (green). The second row shows Flag-tagged p.A287T mutant form of PRRT2 (red), live-labeling of the surface HA-tagged GRIA1 (green). The last row shows Flag-tagged p.R217Pfs*8 mutant form of PRRT2 (red), live-labeling of the surface HA-tagged GRIA1 (green). Scale bar, 10 μm. DAPI (blue) was used to show nuclei; (**B**) Histogram shows the fold change of membrane GRIA1 intensity. Values are represented as mean ± SD, *n =* 10, ** *p* < 0.01.

SYNDIG1 and PRRT2 belong to the large gene family of TM proteins termed Dispanins [21]. Syndig1 has been shown to interact with Gria2 and changed the distribution of Gria2 on the cell membrane [22]. To determine whether PRRT2 alters GRIA1 distribution in our experiments, we transfected COS-7 cells with HA-tagged GRIA1 and either the wild type or mutant Flag-tagged PRRT2 vectors. We used anti-HA antibody to live-label the GRIA1 surface, and anti-Flag antibody to assess the distribution of PRRT2 after the cells were fixed and permeabilized. We revealed wild type PRRT2 to be mainly located at the membrane, while PRRT2 with the missense mutation p.A287T was dispersed in the cytoplasm (Figure 3E,F, respectively). We did not observe any obvious signals with the truncated PRRT2 cells (Figure 5A). However, compared with wild type, surface-labeled HA-GRIA1 increased upon co-expression with mutant PRRT2 (Figure 5A,B), suggesting that mutant PRRT2 not only affects the surface distribution of GRIA1, but may also influence AMPA receptor function.

## 3. Discussion

PRRT2 mutations are involved in many disorders including PKC, benign familial infantile epilepsy (BFIE, MIM 605751), infantile convulsions with choreoathetosis (ICCA, MIM 602066) [23], hemiplegic and regular migraines, paroxysmal non-kinesigenic dyskinesia (PNKD), paroxysmal exertion-induced dyskinesia (PED), childhood absence epilepsy, febrile seizures both in family cases [24] and sporadic cases [25] and episodic ataxia [26]. Meanwhile, homozygous PRRT2 mutations have been more frequently associated with intellectual disabilities [23,26,27]. These findings heavily implicate PRRT2 as a pleiotropic gene, which means defining the function of PRRT2 can serve to elucidate the underlying mechanisms for a broad range of different disorders.

Here, for the first time, we report higher levels of EAAs in the plasma of PKC patients. Consistent with earlier studies that demonstrated a significant correlation between CSF and plasma concentrations for glutamate [17,18], we detected higher glutamate levels in the culture medium of neurons following Prrt2 knock down gene expression. Our results suggest increased glutamate in the CSF of PKC patients could lead to neuronal hyperexcitability. In fact, high extracellular glutamate concentrations in the brain have been identified as a likely trigger of epileptic seizures in mesial temporal lobe epilepsy (MTLE) [13,28]; these might be associated with decreased hippocampal volumes [29]. By studying genetic absence epilepsy rats from Strasbourg (GAERS), Sirvanci *et al.* [30] found that the glutamate density in the CA3 region of GAERS hippocampus was significantly increased compared to the control group. Podell and Creey *et al.* [31,32] found that high glutamate levels in the CSF of idiopathic epilepsy dogs developed between the first observed seizure and CSF sample collection independent of time. Meldrum pointed out that an increase in the extracellular concentrations of glutamate and aspartate in the brain before or during seizure onset suggested enhanced amino acid release, and glutamate antagonists are potent anticonvulsants in animal models of epilepsy [33]. Moreover, an excellent response to add-on lamotrigine treatment may be characterized by decreased plasma glutamate levels, which indicated that the CSF glutamate concentration might also be lower while the drug is circulating [34]. On the other hand, many investigations have shown that in the plasma and CSF of patients with migraine, glutamate levels are increased. Ferrari *et al.* [35] found that migraine patients had higher levels of serum glutamate and aspartate than healthy controls or patients with tensional headache. Cananzi *et al.* [36] mentioned that increased glutamate concentrations were found in the plasma and platelets of migraine patients with and without aura. High CSF glutamate levels have also been reported in patients with chronic daily headache [37], and in chronic migraine patients with or without fibromyalgia [38]. More interestingly, chronic migraine patients who overuse triptans showed lower glutamate levels in CSF [39]. Effective prophylactic treatments of migraine work by decreasing plasma glutamate levels [40]. Although glutamate is not thought to readily cross the blood-brain barrier (BBB), the permeability of the BBB can be changed. Based on all of the previous studies and our current results where we observed high levels of glutamate both in the plasma of PKC patients and in the culture medium of neurons after knocked down the expression of Prrt2, we cannot deny the positive relationship between plasma and CSF but transport of glutamate via the BBB remains a challenging problem. Isoflurane significantly increased the plasma and CSF glutamate level, probably through changing the permeability of the BBB [41]. Stress could be a trigger factor for PKC [2], and can also increase the permeability of the BBB [42]. Even the high concentration of glutamate itself can disrupt the BBB [43]. This implicates interplay between CSF and plasma glutamate levels. In one study, a mouse model of PNKD showed increased dopamine release to caffeine and higher expression of dopamine receptors, indicating the dysfunction of dopamine signaling induces dyskinesias [44]. However, no biogenic amine abnormalities have been reported in CSF analysis of PKC patients [45]. Although we observed significantly higher levels of EAAs in the plasma of PKC patients, our sample size was small. Therefore, the relationship between higher levels of EAAs in the plasma and PKC must be investigated in further experiments with larger sample sizes.

In our wild type experiments, we confirmed interactions between PRRT2 and SNAP25, which inhibited glutamate release and avoided neuronal hyperexcitability. We found that the missense mutant PRRT2 lost its membrane location and weakened its interaction with SNAP25. The decreased or even lost interactions between mutant PRRT2 and SNAP25 potentially resulted in the increased release of glutamate. It has already been reported that 5-HT GPCRs releasing G protein mediate presynaptic inhibition [19]. Prashant *et al.* [46] reported that adding the specific SNAP25 antibody to lysed synaptosomal membranes blocked the released of glutamate, while the normal IgG did not have the same effect. BoNT/E exerts antiepileptic effects and delays neuronal death by inhibiting glutamate release [12,47], while BoNT/A has also been shown to reduce chronic migraine symptoms in clinical trials [48]. Both specifically cleave SNAP25 to inhibit neuronal exocytosis. This may well explain the pleiotropic outcomes of PRRT2 mutations, however, further studies are needed to determine more detailed mechanisms of how PRRT2 influences the function of SNAP25.

In this study, we also proved interactions between PRRT2 and GRIA1. We found that wild type PRRT2 limits the membrane distribution of GRIA1. The increased distribution of GRIA1 on the membrane has been reported in different epilepsy models [49,50]. Kennard *et al.* [49] found increased plasma membrane GRIA1 in the somatosensory cortex neurons of adult epileptic GAERS, which potentially contributes to hyperexcitability in the somatosensory cortex. Rajasekaran *et al.* [50] reported that strengthening of AMPA receptor-mediated neurotransmission in CA1 pyramidal neurons during established status epilepticus is associated with an increased surface expression of GRIA1. Common variants in the regulatory regions of the *GRIA1* gene showed a strong association with migraine patients with and without aura [51]. Both competitive and noncompetitive AMPA receptor antagonists have been used in clinical trials as drugs for epilepsy and migraine patients [52,53]. Therefore, blocking glutamatergic transmission may in fact benefit patients with PRRT2-related disorders. Importantly, PKC patients with PRRT2 mutations have always responded completely to low-dose carbamazepine, while 94% of PKC patients without PRRT2 mutations did not have a good response to carbamazepine, even after the dose of the drug was increased [54].

**Figure 6:**
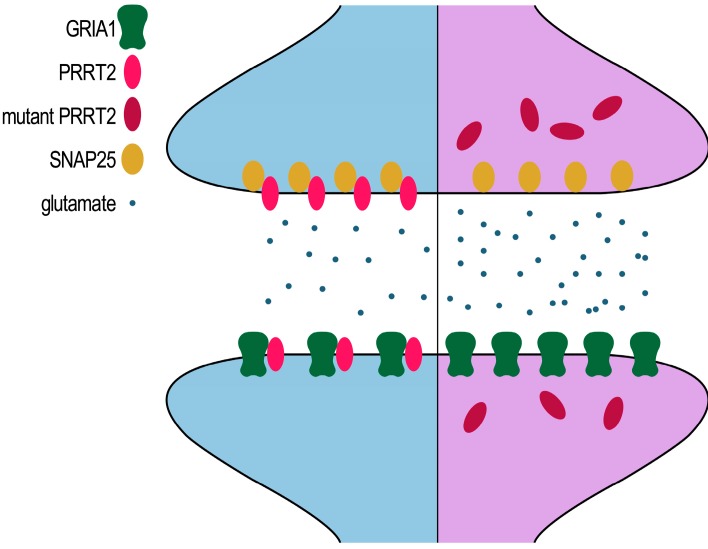
Schematic representation of mutant PRRT2 affecting the glutamate signaling pathway.

Taken together, our results indicate that the release of glutamate and the function of ionotropic glutamate receptors were inferred by mutations to PRRT2, ultimately leading to neuronal hyperexcitability (Figure 6). As PRRT2 is a novel gene involved in modulating neuronal excitability, further investigations are needed to provide new insights into understanding the mechanisms of PRRT2 related disorders and offer a new perspective for clinical therapy.

## 4. Experimental Section

### 4.1. Chemicals and Antibodies

Paraformaldehyde (PFA), *O*-phthaldialdehyde (OPA), l-aspartic acid, l-glutamic acid and glycine were all purchased from Sigma-Aldrich (St. Louis, MO, USA). Potassium acetate was purchased from Sinopharm Chemical Reagent Co., Ltd. (Shanghai, China). Methanol was purchased from Merck, Darmstadt, Germany. Phenylmethanesulfonyl fluoride (PMSF) and Triton X-100 were purchased from Amresco (Solon, OH, USA). We used the following antibodies: rabbit anti-GAPDH IgG, mouse anti-HA IgG, mouse and rabbit anti-Myc IgG (CWBio, Beijing, China), rabbit anti-Flag IgG (Cell Signaling Technology, Danvers, MA, USA), rabbit anti-GRIA1 IgG, mouse anti-PSD95 IgG (Millipore, Bedford, MA, USA), goat anti-vGlut1 IgG (Santa Cruz Biotechnology, Santa Cruz, CA, USA), mouse anti-Flag IgG, rabbit anti-PRRT2 IgG, poly-d-lysine (PDL) and 0.25% trypsin (Sigma-Aldrich, St. Louis, MO, USA). We purchased normal mouse IgG from Santa Cruz Biotechnology (Santa Cruz, CA, USA). Secondary antibodies for immunofluorescence studies were Alexa 488 donkey anti-rabbit and anti-mouse IgG, Alexa 594 donkey anti-mouse, anti-goat and anti-rabbit IgG (Invitrogen, Carlsbad, CA, USA). We used the following secondary antibodies for western blotting: horseradish peroxidase-conjugated goat anti-rabbit, goat anti-mouse and rabbit anti-goat IgG (CWBio). We purchased donkey serum from Invitrogen (Carlsbad, CA, USA).

### 4.2. Subjects and Plasma Preparation

We picked seven patients of Han Chinese descent, all diagnosed with PKC according to the clinical diagnostic criteria [2] at the Neurology department of Xuanwu hospital. We recruited 12 matched healthy control subjects from Peking Union Medical College Hospital. The local hospital medical ethics committee approved this study. All participating subjects fasted from 8:00 pm. On the next day, we collected blood samples from all participating subjects between 8:00 and 9:00 am after informed consent was obtained. We separated plasma and stored the samples at −80 °C. Prior to HPLC analysis, we purified the plasma as previously described [55].

### 4.3. Animals

Animal care and experimental protocols were in compliance with Experimental Animal Regulations (China Science and Technology Commission Order No. 2) and approved by the Institutional Animal Care and Use Committee at the Institute of Basic Medical Sciences, Chinese Academy of Medical Sciences and Peking Union Medical College. For our immunofluorescence studies, adult Institute of Cancer Research (ICR, London, UK) mouse brains were fixed in 4% PFA for 12 h, dehydrated in phosphate-buffered saline, (PBS pH 7.4) containing 20% sucrose until the tissue sank and embedded in SAKURA Tissue-Tek O.C.T. Compound (SAKURA, Torrance, CA, USA). Frozen tissue samples were sectioned (16 μm thick) and stored at −80 °C until required.

### 4.4. Aspartate, Glutamate and Glycine Content Measurement

We determined the concentrations of aspartate, glutamate and glycine by HPLC using an Athena C18-WP column (4.5 × 150 mm, 5 μm, ANPEL Scientific Instrument Co., Ltd., Shanghai, China) in a Waters (Milford, MA, USA) Alliance consisting of a 2690 separation model and 996 Photodiode Array after pre-column derivatization with OPA at an emission wavelength (λem) of 338 nm [56]. We used 50 mM sodium acetate buffer for mobile phase A and methanol for mobile phase B. The gradient elution procedure was: 0 to 1 min, A-B (85:15, *v*/*v*); 1 to 6 min, A-B (55:45, *v*/*v*); 6 to 15 min, A-B (55:45, *v*/*v*); 15 to 21 min, A-B (25:75, *v*/*v*); 21 to 25 min, A-B (10:90, *v*/*v*); 25 to 29 min, A-B (85:15, *v*/*v*) and 29 to 30 min, A-B (85:15, *v*/*v*). The flow rate was 0.8 mL/min. 0.001 M aspartate, glutamate and glycine stock solutions were diluted to different concentrations before use as standards.

### 4.5. Cell Culture and Transfection

HEK293T cells and COS-7 cells obtained from ATCC were cultured in Dulbecco’s modified Eagle medium (DMEM) with 10% fetal bovine serum (Invitrogen, Carlsbad, CA, USA) and transfected using Lipofectamine 2000 (Invitrogen, Carlsbad, CA, USA) according to manufacturer’s instructions. E18.5 ICR mouse cortex primary neurons were isolated and maintained in Neurobasal-A medium (Invitrogen) with B27 (Invitrogen) as previously reported [57]. Briefly, cerebral cortices were removed from embryos stripped of meninges and vessels. After that, tissues were minced and digested by 0.25% trypsin for 5 min at 37 °C triturated gently. Neurons were cultured on 12-well plates coated with PDL (50 μg/mL) at a density of 3 × 10^5^ cells/cm^2^. Cells were cultured in a humidified atmosphere with 5% CO_2_ and, when performing immunofluorescence experiments (Figure S2), plated onto glass slips (Thermo Scientific, Rockford, IL, USA).

### 4.6. Plasmid Constructions

We used human brain total mRNA (Clontech, Mountain View, CA, USA) for the amplification of PRRT2, SNAP25 and GRIA1. We obtained cDNA from mouse brain for Prrt2 and Gria1 amplification. Briefly, we utilized the *Eco*RI and *Sal*I sites of the pCMV-Myc vector (Clontech, Mountain View, CA, USA) to clone the full-length Prrt2. Full-length Gria1 was cloned into the *Eco*RI and *Xho*I sites of the pCMV-HA vector (Clontech) with a HA-tag after the *C*-terminal. The *Sal*I and *Kpn*I sites of pCMV-Myc vector (Clontech) were used for the cloning full-length SNAP25. Full-length GRIA1 was cloned in to the *Eco*RI and *Kpn*I sites of pCMV-HA vector (Clontech, Mountain View, CA, USA) with a HA-tag insert after the signal peptide. Human wild type PRRT2 full-length was cloned into the *Xba*I and *Bam*HI sites of the pFLAG-CMV-2 vector (Sigma-Aldrich, St. Louis, MO, USA). For generating PRRT2 c.859G>A (PRRT2 p.A287T), we performed site-directed mutagenesis by PCR using the wild type vector. We synthesized the full-length sequence of the PRRT2 c.649_650insC (PRRT2 p.R217Pfs*8) and cloned it into the same site of the pFLAG-CMV-2 vector. All primers (Table S2) and the truncated mutant PRRT2 (Table S3) were synthesized by Sangon Biotech (Shanghai, China).

### 4.7. Recombinant Lentivirus Infection

BLOCK-iT RNAi Designer (Invitrogen, Carlsbad, CA, USA) was used to predict the shRNA sequence (5'-TGCCAGCATCCAAACCAGATGTTTCAAGAGAACATCTGGTTTGGATGCTGGCTTTTTTC-3') of targeted mouse Prrt2. The shRNA sequence was cloned into the *Hpa*I and *Xho*I sites of the pLL3.7 vector (Shanghai CPG Biotech Co., Ltd., Shanghai, China). Western blotting was used to test the gene silencing efficiency. Recombinant lentivirus was prepared by co-transfection pLL3.7 or recombined pLL3.7 vectors and packaging vectors into HEK293T cells as previously described [58]. Primary cultured mouse cortex neurons at day six were incubated with lentivirus supernatant for 8 h before changing into fresh medium. After 96 h of infection, the culture medium was collected and stored at −80 °C. Prior to HPLC analysis, the medium was purified as previously reported [55,59].

### 4.8. Co-Immunoprecipitation

HEK293T cells were co-transfected with the appropriate vectors and lysed after 48 h using co-immunoprecipitation assay lysis buffer (Bio TeKe Corporation, Beijing, China) and 1 mM PMSF. Equal amounts of lysate from cells or mouse brains were incubated with primary antibody or normal mouse IgG overnight at 4 °C and then with Protein A/G PLUS-Agarose (Santa Cruz Biotechnology) for 3 h. We used lysates without any treatment as input controls. Co-immunoprecipitation protocols were in accordance with the manufacturer’s instructions (sc-2003, Santa Cruz Biotechnology). All samples were denatured before western blotting analysis.

### 4.9. Western Blotting

Equal amounts of protein samples were separated by SDS-PAGE and then transferred onto nitrocellulose membrane (Millipore, Bedford, MA, USA), which was followed by blocking with 5% nonfat milk (Sangon Biotech, Shanghai, China) and diluting in tris-buffered saline with 0.1% Triton X-100 (TBST) for 1 h at room temperature (RT). Later, the membrane was incubated with primary antibodies (anti-PRRT2 IgG: 1:1000; anti-vGlut1 IgG: 1:1000; anti-GAPDH IgG: 1:5000; anti-Flag IgG: 1:1000; anti-Myc IgG: 1:1000; anti-HA IgG: 1:1000; anti-Gria1 IgG: 1:1000) overnight at 4 °C and washed with TBST. This was followed by incubation with horseradish peroxidase-conjugated secondary antibodies for 1 h at RT. Blot signals were then detected by Chemistar High-sig ECL Western Blotting Substrate (Tanon, Shanghai, China). We quantified the absolute intensity of the bands using the IMAGEJ 1.48 software (National Institutes of Health, Boston, MA, USA). Protein levels after immunoprecipitation were normalized against the protein they interacted with (either Flag-tagged PRRT2 or HA-tagged GRIA1) and compared with the wild type group.

### 4.10. Immunofluorescence Experiments

COS-7 cells transfected with the appropriate plasmids were fixed in 100% methanol for 10 min at −20 °C. Frozen sections were fixed in 4% PFA for 10 min at RT. After fixation, cells or the frozen sections were rinsed with PBS, permeabilized and blocked with PBS containing 0.3% Triton X-100 and 5% donkey serum for 30 min. Afterwards, they were incubated with primary antibodies (anti-Prrt2 IgG: 1:50; anti-vGlut1 IgG: 1:50; anti-PSD95 IgG: 1:200; anti-Flag IgG: 1:200) and washed, which was followed by incubation with secondary antibodies. Finally, they were mounted with mounting medium containing 4',6-diamidino-2-phenylindole (DAPI) (Zhongshan Goldenbridge Biotechnology, Beijing, China). All images were captured using Olympus FV1000 confocal microscopy (Tokyo, Japan). GRIA1 intensity was analyzed using the IMAGEJ 1.48 software (National Institutes of Health).

### 4.11. Live-Labeling

For live-labeling, cells were first labeled by the primary antibody (anti-HA IgG: 1:200) diluted in warm culture medium without fetal bovine serum for 30 min at 37 °C. Thereafter, cells were washed by warm culture medium without fetal bovine serum and incubated with the secondary antibody Alexa 488 donkey anti-mouse IgG diluted in culture medium without fetal bovine serum. After a final rinse with culture medium without fetal bovine serum, cells were fixed and incubated with additional primary antibodies as mentioned above [22].

### 4.12. Statistical Analysis

Amino acids concentrations were compared between groups using one-way ANOVA. Student’s *t*-tests were used to analyze all of the other experiments. *p* < 0.05 was considered significant. Quantitative values are shown as mean ± SD.

## 5. Conclusions

In the present study, we found higher glutamate levels in the plasma of PKC patients and in the culture medium of neurons when Prrt2 expression was knocked down. We further found that the interaction of PRRT2 with SNAP25 was disturbed by the missense mutation of PRRT2. At last, we proved the interaction between PRRT2 and GRIA1 for the first time. Moreover, the membrane distribution of GRIA1 was increased when interacted with the mutant forms of PRRT2.

## Supplementary Information

Supplementary materials can be found at: http://www.mdpi.com/1422-0067/16/05/9134/s1.
