# Supplementary material for: PRRT2 Mutant Leads to Dysfunction of Glutamate Signaling
Source: Int J Mol Sci. 2015 Apr 23;16(5):9134–51. doi: 10.3390/ijms16059134 (PMC4463582; doi:10.3390/ijms16059134)
Supplement: Supplementary file 1 [file ijms-16-09134-s001.pdf]

# Supplementary Information

Figure S1. Methods: HEK293T cells obtained from ATCC were cultured in Dulbecco's modified Eagle medium (DMEM) with 10% fetal bovine serum (Invitrogen, Carlsbad, CA, USA) and transfected using Lipofectamine 2000 (Invitrogen, Carlsbad, CA, USA) according to the manufacturer's instructions. Cells were co-transfected with pCMV-Prrt2 and shRNA-Prrt2 plasmids for 48 h. After that, total RNA was extracted with TRIZOL (CWBio, Beijing, China) according to the manufacturer's instructions. 1 μg of total RNA was used for reverse transcription by ReverTra Ace qPCR RT Kit (TOYOBO, Osaka, Japan) following the manufacturer's instructions. All qPCR reactions were performed in triplicate using the UltraSYBR Mixture (with ROX; CWBio, Beijing, China) according to the manufacturer's instructions. Total protein was extracted and equal amounts of protein samples were separated by SDS-PAGE and then transferred onto the nitrocellulose membrane (Millipore, Bedford, MA, USA), which was followed by blocking with 5% nonfat milk (Sangon Biotech, Shanghai, China) and diluting in TBST for 1 h at room temperature (RT). Later, the membrane was incubated with primary antibodies (anti-Myc IgG: 1:1000; anti-GAPDH IgG: 1:5000) overnight at 4°C and washed with TBST, which was followed by incubation with horseradish peroxidase-conjugated secondary antibodies for 1 h at RT. Blot signals were detected by Chemistar High-sig ECL Western Blotting Substrate (Tanon, Shanghai, China). Rabbit anti-GAPDH and mouse anti-Myc antibody were purchased from CWBio, Beijing, China.

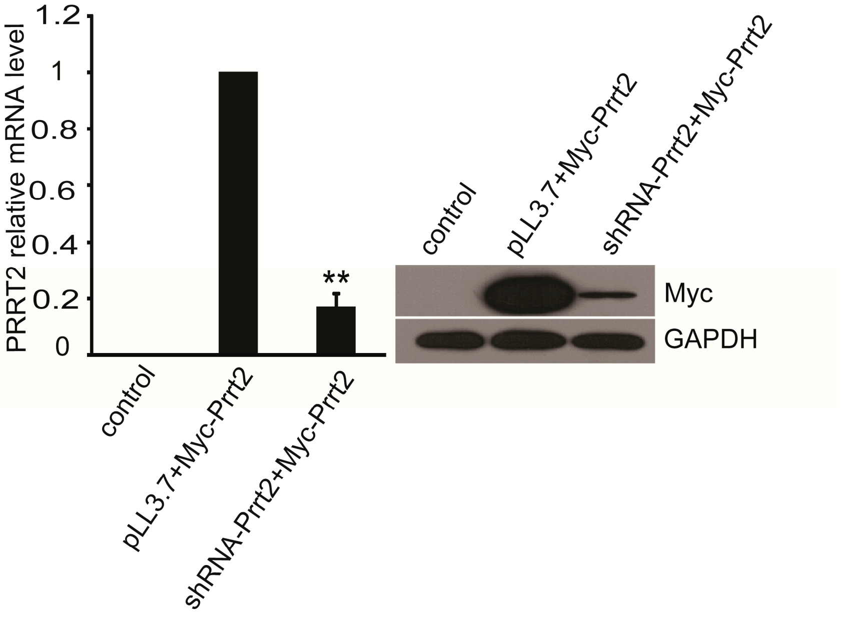


**Figure S1.** shRNA-Prrt2 efficiently knocked down Prrt2 expression. Prrt2 mRNA and protein levels were detected by real time PCR and western blotting, ** $p < 0.01$.

Figure S2. Methods: E18.5 ICR mouse cortex primary neurons were isolated and maintained in Neurobasal-A medium (Invitrogen, Carlsbad, CA, USA) with B27 (Invitrogen, Carlsbad, CA, USA). Briefly, cerebral cortices were removed from embryos stripped of meninges and vessels. After that, tissues were minced and digested with 0.25% trypsin for 5 min at 37 °C triturated gently. Neurons were cultured on glass slips coated with PDL (50 μg/mL) at a density of $3 \times 10^5$ cells/cm$^2$. At DIV 8, the neurons were fixed in 100% methanol for 10 min at −20 °C. After fixation, cells were rinsed in PBS,

permeabilized and blocked with PBS containing 0.3% Triton X-100 and 5% donkey serum for 30 min. Afterward, they were incubated with anti-MAP2 antibodies (Sigma-Aldrich, St. Louis, MO, USA) and washed, incubated with Alexa 488 donkey anti-mouse IgG (Invitrogen, Carlsbad, CA, USA). Finally, they were mounted with mounting medium containing 4',6-diamidino-2-phenylindole (DAPI) (Zhongshan Goldenbridge Biotechnology, Beijing, China). All images were captured using an Olympus FV1000 confocal microscope (Tokyo, Japan).

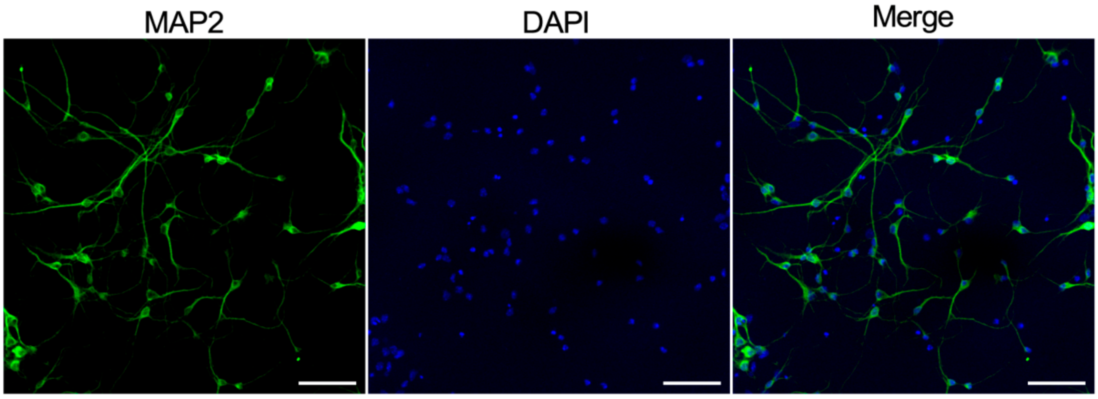


**Figure S2.** Characterization of primary cultured mouse cortex neurons. Immunostaining of DIV 8 neurons show the dendritic marker MAP2 (green); DAPI (blue) indicates the nucleus. Scale bar, 50 μm.

**Table S1.** Primers for real-time PCR.

| **Primers** | **Primer Sequences (5'-3')** |
|---|---|
| Prrt2-F | TCGGGACTATATCATCCTTGCC |
| Prrt2-R | AGCGCCACGATGCTTAAGAG |
| GAPDH-F | CTGCACCACCAACTGCTTAG |
| GAPDH-R | GTGGATGCAGGGATGATGTTC |

F, forward primer; R, reverse primer.

**Table S2.** Primers for plasmids construction. Sequences are from 5' to 3'.

| | |
|---|---|
| **Primers for full-length mouse *Prrt2*** | |
| mPrrt2-F | CCGGAATTCCTCTCCCATCTCGCTTCTCT |
| mPrrt2-R | ACGCGTCGACTCCCAACAGGAAGAAAAGTC |
| **Primers for full-length mouse *Gria1*** | |
| mGria1-F | CCGGAATTCAAAGGAATATGCCGTACATC |
| mGria1-R | CCGCTCGAGTTAAGCGTAATCTGGAACATCGTATGGGTACAATCCTGTGGCTCCCAA |
| **Primers for full-length human *SNAP25*** | |
| hSNAP25-F | ACGCGTCGACCATGGCCGAAGACGCAGACA |
| hSNAP25-R | CGGGGTACCACGGGTGGGCACACTTAACC |
| **Primers for full-length human *GRIA1*** | |
| hGRIA1-F | CCGGAATTCATGCAGCACATTTTTGCCTTCTTCTGCACCGGTTTCCTAGGCGCGGTAGT AGGTGCCTACCCATACGATGTTCCAGATTACGCTAATTTCCCCAACAATATCCA |
| hGRIA1-R | CGGGGTACCCAGTTACAATCCCGTGGCTC |
| **Primers for full-length wild type human *PRRT2*** | |
| hPRRT2-F | CTAGTCTAGAATGGCAGCCAGCAGCTCTG |
| hPRRT2-R | CGCGGATCCCTTAGGCGTGTATAAGTGA |
| **Primers for full-length human *PRRT2* with c.859G>A mutation** | |
| mut-F1 | CCCTCTCCCATCTCAAG |
| mut-R1 | ATAAGCGAAGGTCACGATGTTGACA |
| mut-F2 | AACATCGTGACCTTCGCTTAT |
| mut-R2 | TCCCAACAGGAAGAAAAGT |

F, forward primer; R, reverse primer.

**Table S3.** The full-length sequence of *PRRT2* c.649_650insC.

| **The Sequence of the *PRRT2* c.649_650insC** |
|---|
| AAGCTTATGGCAGCCAGCAGCTCTGAGATCTCTGAGATGAAGGGGGTTGAGGAGAGTCCCA AGGTTCCAGGCGAAGGGCCTGGCCATTCTGAAGCTGAAACTGGCCCTCCCCAGGTCCTAGC AGGGGTACCAGACCAGCCAGAGGCCCCGCAGCCAGGTCCAAACACCACTGCGGCCCCTGT GGACTCAGGGCCCAAGGCTGGGCTGGCTCCAGAAACCACAGAGACCCCGGCTGGGGCCTC AGAAACAGCCCAGGCCACAGACCTCAGCTTAAGCCCAGGAGGGGAATCAAAGGCCAACTG CAGCCCCGAAGACCCATGCCAAGAAACAGTGTCCAAACCAGAAGTGAGCAAAGAGGCCAC TGCAGACCAGGGGTCCAGGCTGGAGTCTGCAGCCCCACCTGAACCAGCCCCAGAGCCTGCT CCCCAACCAGACCCCCGGCCAGATTCCCAGCCTACCCCCAAGCCAGCCCTTCAACCAGAGC TCCCTACCCAGGAGGACCCCACCCCTGAGATTCTGTCTGAGAGTGTAGGGGAAAAGCAAGA GAATGGGGCAGTGGTGCCCCTGCAGGCTGGTGATGGGGAAGAGGGCCCAGCCCCTGAGCC TCACTCACCACCCTCAAAAAAATCCCCCCCAGCCAATGGGGCCCCCCCCCCGAGTGCTGCA GCAGCTGGTTGAGAATTC |
